# *Streptococcus pneumoniae* TIGR4 Phase-Locked Opacity Variants Differ in Virulence Phenotypes

**DOI:** 10.1128/mSphere.00386-17

**Published:** 2017-11-15

**Authors:** Melissa B. Oliver, Ankita Basu Roy, Ranjit Kumar, Elliot J. Lefkowitz, W. Edward Swords

**Affiliations:** a Department of Medicine, Division of Pulmonary, Allergy, and Critical Care Medicine, University of Alabama at Birmingham, Birmingham, Alabama, USA; b Gregory Fleming James Cystic Fibrosis Research Center, University of Alabama at Birmingham, Birmingham, Alabama, USA; c Biomedical Informatics, Center for Clinical and Translational Sciences, University of Alabama at Birmingham, Birmingham, Alabama, USA; d Department of Microbiology, University of Alabama at Birmingham, Birmingham, Alabama, USA; University of Kentucky

**Keywords:** *Streptococcus pneumoniae*, phase variation, pneumococcus

## Abstract

*Streptococcus pneumoniae* (pneumococcus) is a leading human pathogen that can cause serious localized and invasive diseases. Pneumococci can undergo a spontaneous and reversible phase variation that is reflected in colony opacity and which allows the population to adapt to different host environments. Generally, transparent variants are adapted for nasopharyngeal colonization, whereas opaque variants are associated with invasive disease. In recent work, colony phase variation was shown to occur by means of recombination events to generate multiple alleles of the *hsdS* targeting domain of a DNA methylase complex, which mediates epigenetic changes in gene expression. A panel of isogenic strains were created in the well-studied *S. pneumoniae* TIGR4 background that are “locked” in the transparent (*n* = 4) or opaque (*n* = 2) colony phenotype. The strains had significant differences in colony size which were stable over multiple passages *in vitro* and *in vivo*. While there were no significant differences in adherence for the phase-locked mutant strains to immortalized epithelial cells, biofilm formation and viability were reduced for the opaque variants in static assays. Nasopharyngeal colonization was stable for all strains, but the mortality rates differed between them. Transcript profiling by transcriptome sequencing (RNA-seq) analyses revealed that the expression levels of certain virulence factors were increased in a phase-specific manner. As epigenetic regulation of phase variation (often referred to as "phasevarion") is emerging as a common theme for mucosal pathogens, these results serve as a model for future studies of host-pathogen interactions.

**IMPORTANCE** A growing number of bacterial species undergo epigenetic phase variation due to variable expression or specificity of DNA-modifying enzymes. For pneumococci, this phase variation has long been appreciated as being revealed by changes in colony opacity, which are reflected in changes in expression or accessibility of factors on the bacterial surface. Recent work showed that recombination-generated variation in alleles of the HsdS DNA methylase specificity subunit mediated pneumococcal phase variation. We generated phase-locked populations of *S. pneumoniae* TIGR4 expressing a single nonvariant *hsdS* allele and observed significant differences in gene expression and virulence. These results highlight the importance of focused pathogenesis studies within specific phase types. Moreover, the generation of single-allele *hsdS* constructs will greatly facilitate such studies.

## Abstract (precis)

A growing number of bacterial species undergo epigenetic phase variation due to variable expression or specificity of DNA-modifying enzymes. For pneumococci, this phase variation has long been appreciated as being revealed by changes in colony opacity, which are reflected in changes in expression or accessibility of factors on the bacterial surface. Recent work showed that recombination-generated variation in alleles of the HsdS DNA methylase specificity subunit mediated pneumococcal phase variation. We generated phase-locked populations of *S. pneumoniae* TIGR4 expressing a single nonvariant *hsdS* allele and observed significant differences in gene expression and virulence. These results highlight the importance of focused pathogenesis studies within specific phase types. Moreover, the generation of single-allele *hsdS* constructs will greatly facilitate such studies.

## INTRODUCTION

*Streptococcus pneumoniae* (pneumococcus) is an opportunistic pathogen that often resides in the human nasopharynx of healthy individuals (1–3). Nasopharyngeal colonization invariably precedes development of invasive disease (e.g., pneumonia, sepsis, and meningitis) in susceptible individuals, such as young children and older adults (4–7). Pneumococcal populations undergo spontaneous and reversible intrastrain phase variation between opaque and transparent colony phenotypes (8–11). Although a strain of pneumococcus can exist as a combination of the two phenotypes (12), the opaque forms express more capsule (13), particularly under anaerobic conditions (e.g., in blood) (10); have increased resistance to opsonophagocytosis and host clearance; and are associated with invasive disease (8, 12–14). The transparent forms have higher levels of exposed cell wall teichoic acid (11), increased representation in biofilms (15–18), and increased adherence to human epithelial cells (14); show efficient colonization of the nasopharynx (8, 9); and are generally considered noninvasive. Phase variation is thought to facilitate adaptation during different stages of infections, such as dissemination from the nasopharynx into the lung (9).

A major limitation in the field of pneumococcal phase variation research is the lack of genetically “locked” strains that are incapable of switching phenotype. Typical studies use bacterial populations enriched for each phenotype via selection of a single colony and serial passage until the majority of colonies are uniformly one phenotype (8). While useful, such strains are not genetically “locked” into a particular phase and can freely switch back and forth. Recent work has shown that pneumococcal phase variation of colony opacity occurred via site-specific recombination of the three methylase sequence specificity genes (*hsdS*, *hsdS*′, and *hsdS*′′) in the SpnD39III and Spn556II type I restriction-modification (R-M) systems (19, 20). Specifically, recombination of the three genes produced six predicted *hsdS* alleles that can generate six bacterial subpopulations with distinct colony phenotypes, virulence, DNA methylation patterns, and changes in gene expression (19, 20).

Like many other bacteria, pneumococci encode multiple R-M systems to provide a key defense mechanism against invasion of foreign DNA (e.g., bacteriophage DNA) and protection of host DNA (via methylation) from endogenous restriction enzyme cleavage. The pneumococcal SpnD39III and Spn556II type I R-M systems include genes *hsdS*, *hsdM*, and *hsdR* and a site-specific recombinase gene. The *hsdS*, *hsdM*, and *hsdR* genes are cotranscribed into protein subunits HsdS, HsdM, and HsdR, which assemble into a heteromeric enzyme that can methylate and cleave double-stranded DNA. The HsdS subunit is the determinant of DNA methylase sequence specificity and has two different DNA target recognition domains separated by conserved regions that recognize “split” sequences (e.g., 5′-CRAAnnnnnnnnCTG-3′) (19). The HsdM subunit is responsible for DNA methylation at the appropriate adenine within the DNA recognition sequence (see the underlined adenine in the sequence example). The HsdR subunit is responsible for DNA cleavage and translocation through the bound heteromeric complex (21). This *hsd* type I R-M system is used by many bacterial pathogens, including *Escherichia coli*, *Mycoplasma pneumoniae*, *Staphylococcus aureus*, *S. pneumoniae*, and others (19, 22–24). In this study, we sought to assess the role that the six predicted *hsdS* alleles might play in colony phenotype, gene expression, and pathogenesis using a mouse model for nasal colonization. The *S. pneumoniae* TIGR4 genetic background strain was chosen since it is a commonly used model strain for which genomic sequence data are available (25).

## RESULTS

### Pneumococcal *hsdS* genetic loci.

**FIG 1:**
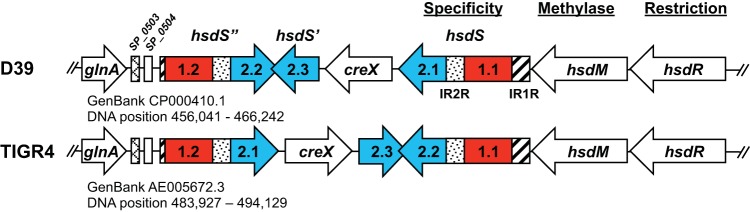
Homology comparison of the type I restriction modification loci from strains D39 and TIGR4. Comparative analyses of the published *hsdS* alleles (GenBank accession numbers KJ955483 to KJ955486, KJ398403, and KJ398404, corresponding to alleles A to D, E, and F, respectively) determined the coding sequences for each TRD. Both strains have the six TRDs (shown in red and blue) that can be rearranged to generate six predicted *hsdS* alleles. Restriction by HsdR and DNA methylation by HsdM are specified by the TRDs present in *hsdS*. IR1R/IR2R, inverted repeat 1/2; *creX*, recombinase.

Examination of the six published *hsdS* allele sequences (A, B, C, D, E, and F, corresponding to GenBank accession numbers KJ955483, KJ955484, KJ955485, KJ955486, KJ398403, and KJ398404, respectively) and comparative analyses of their translated protein sequences led us to define the coding sequence for each HsdS target recognition domain (TRD). The pneumococcal strain D39 HsdS protein was divided into four sections: inverted repeat 1R (IR1R; codons 1 to 92), TRD 1.1 (codons 93 to 232), IR2R (codons 234 to 336), and TRD 2.1 (codons 337 to 522). With this information in hand, we annotated the homologous *hsdS* genetic locus in pneumococcal strain TIGR4 (GenBank accession number AE005672.3) (see Table S1 in the supplemental material). Like *S. pneumoniae* D39, *S. pneumoniae* TIGR4 harbored all the TRDs necessary to produce the six predicted *hsdS* alleles (Fig. 1), and their DNA sequences were 100% identical. The only coding mutation identified was a single amino acid substitution (Ile11Val) in the IR1R region of the *hsdS* gene. The orientations and locations for TRD 2.1, TRD 2.2, TRD 2.3, and the site-specific recombinase *creX* differed in *S. pneumoniae* TIGR4. Additionally, the last 47 bp of TRD 2.3 overlapped the last 47 bp of the inverted TRD 2.2 sequence, making mutational studies particularly challenging. Examination of other published pneumococcal genomes revealed that the six TRDs identified in pneumococcal strain D39 (GenBank accession number CP000410.1) were represented in other strains (data not shown). Their gene orientations and locations were either highly similar to those seen with strain D39 (e.g., strain AP200; GenBank accession number CP002121.1) or quite different (e.g., strain 70585; GenBank accession number CP000918.1), highlighting the genetic variability of this region.

### Creation of *S. pneumoniae* TIGR4 *hsdS* variants.

**FIG 2:**
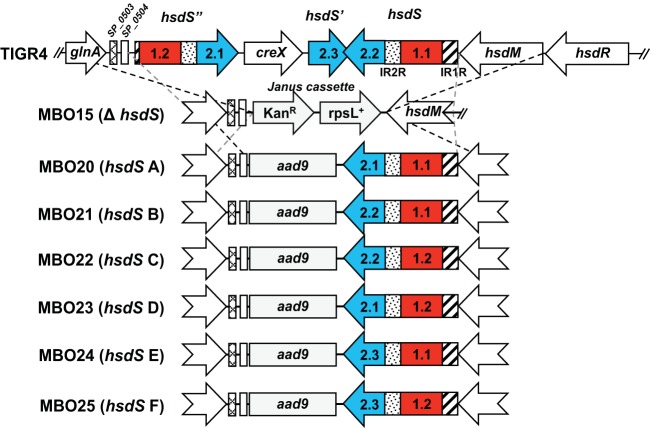
Diagram representing the creation of phase-locked *S. pneumoniae* TIGR4 single-allele *hsdS* mutants. Alleles were produced via overlap extension PCR and cloned adjacent to a selectable marker (spectinomycin resistance gene *aad9*) and to flanking DNA sequences to ensure correct construct integration. Homologous recombination is indicated with dashed lines. Kan^R^, kanamycin resistance.

Strain MBO15 was created by replacing all genes located between *S. pneumoniae* TIGR4 *SP_0504* and *hsdM* with a Janus cassette (26, 27). Overlap extension PCR was used to create the six predicted *hsdS* alleles, which were then cloned in a location adjacent to spectinomycin resistance gene *aad9* and to flanking genes *glnA*, *SP_0503*, and *SP_0504* (Fig. 2). Transformation of each construct into MBO15 produced the recombinant *S. pneumoniae* TIGR4 isogenic mutant strains MBO20, MBO21, MBO22, MBO23, MBO24, and MBO25 containing recombinant *hsdS* alleles A, B, C, D, E, and F, respectively. In this study, strains MBO20 to MBO25 are referred to as strains A to F. All strains used in this study and their associated *hsdS* TRDs are listed in Table 1. Primers used to create the alleles and the knockout construct are listed in Tables S1 and S2.

10.1128/mSphere.00386-17.2 TABLE S1  Summary of coding sequences in the *hsdS* genetic locus. Each gene, target recognition domain (TRD), and inverted repeat (IR1R/IR2R) coding sequence in the TIGR4 *hsdS* genetic locus shown in Fig. 1 has been annotated. DNA positions are based on the TIGR4 sequence (GenBank accession number AE005672.3). Download TABLE S1, DOCX file, 0.01 MB. Copyright © 2017 Oliver et al. 2017 Oliver et al. This content is distributed under the terms of the Creative Commons Attribution 4.0 International license.

10.1128/mSphere.00386-17.3 TABLE S2  Primers used in this study. Hybrid primers F3−F9, R2-R4, and R6 could bind to two different gene targets and were used for overlap extension PCR. For clarity, the DNA binding position and gene targets for both the left side (bolded sequence) and right side (unmodified sequence) of the primers are indicated. DNA positions are based on the TIGR4 sequence (GenBank accession number AE005672.3). Abbreviations: F, forward; R, reverse; IGR, intergenic region; TRD, target recognition domain; IR, inverted repeat 1R/2R; N/A, not applicable. Download TABLE S2, EPS file, 2.9 MB. Copyright © 2017 Oliver et al. 2017 Oliver et al. This content is distributed under the terms of the Creative Commons Attribution 4.0 International license.

**TABLE 1:** List of strains used in this study^a^

Strain name	*hsdS* allele	Phenotype	Comment	Source
TIGR4	TIGR4	Opaque	Clinical isolate (blood)	David E. Briles (UAB)
TIGRJS (NT)	TIGR4	Transparent	TIGR4 *cps*::Janus cassette	David E. Briles (UAB)
MBO15	Δ	Transparent	TIGR4 *hsdS*′ *creX hsdS*::Janus cassette	This study
MBO20	A	Opaque	MBO15 JS::*hsdS* allele A (TRD 1.1 and 2.1)	This study
MBO21	B	Opaque	MBO15 JS::*hsdS* allele B (TRD 1.1 and 2.2)	This study
MBO22	C	Transparent	MBO15 JS::*hsdS* allele C (TRD 1.2 and 2.2)	This study
MBO23	D	Transparent	MBO15 JS::*hsdS* allele D (TRD 1.2 and 2.1)	This study
MBO24	E	Transparent	MBO15 JS::*hsdS* allele E (TRD 1.1 and 2.3)	This study
MBO25	F	Transparent	MBO15 JS::*hsdS* allele F (TRD 1.2 and 2.3)	This study

a Colony phenotypes are indicated. Abbreviations: *cps*, capsular synthesis locus; JS, Janus cassette; TRD, target recognition domain; NT, nontypeable.

### Impact of individual *hsdS* alleles on colony phenotype.

**FIG 3:**
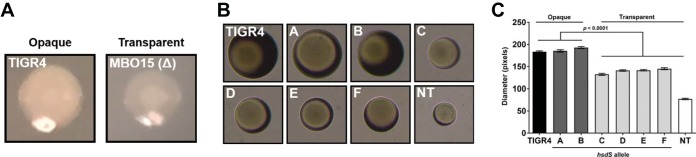
Colony phenotype of single-allele *hsdS* mutants. Colonies of opaque and transparent types of *S. pneumoniae* TIGR4 were visualized under conditions of oblique lighting. (A) Strains were grown on nonselective Trypticase soy agar supplemented with catalase (2,500 U/ml) to a density of less than 100 CFU, harvested, diluted, and passaged for at least five consecutive days to assess population phenotypes by phase-contrast microscopy. Images were taken with a Fisher Micromaster microscope (magnification, ×40). (B) Representative images of colony phenotypes. ImageJ V1.49 was used to measure the diameter of individual CFU, and results are shown as average colony diameters (in pixels) ± standard errors of the means (SEM) of individual CFU. (C) A one-way analysis of variance (ANOVA) performed with a Tukey *post hoc* test determined statistical significance. An unencapsulated TIGR4 strain (TIGRJS; NT) was used as a transparent phenotype control.

Pneumococcal colony phenotypes are determined by visualizing colonies on a translucent agar under oblique lighting (8). Generally, opaque colonies appear as domes of solid color, while transparent colonies appear as colonies that are clear or that have a dense center and translucent halo (Fig. 3A). The colony phenotypes for recombinant *hsdS* strains A to F were determined using strain *S. pneumoniae* TIGR4 and its unencapsulated derivative TIGR-JS as an opaque control and a transparent control, respectively. Briefly, bacterial stocks were grown on nonselective Trypticase soy agar supplemented with catalase for 18 to 20 h. At least 50 colonies were visualized under a dissection microscope, and their phenotypes were recorded. Strains *S. pneumoniae* TIGR4, A, and B had larger, 100% opaque colonies, while strains C, D, E, F, and TIGR-JS had smaller, 100% transparent colonies (Table 1). No mixed populations or colony variants were observed for any of the strains.

In order to quantitatively assess and compare colony phenotypes, at least 100 individual colonies of each strain were visualized with phase-contrast microscopy and photographed and their diameters measured (see Materials and Methods). Within each strain, the colonies were nearly identical in size (Fig. 3B) and had nearly identical diameters (Fig. 3C). Interestingly, the average colony diameters were very similar between the opaque strains *S. pneumoniae* TIGR4, A, and B and also between the transparent strains C, D, E, and F (Fig. 3C). The opaque and transparent colony sizes differed by ~25%. In order to test whether colony phenotypes were stable on passage, the colonies imaged in Fig. 3 were harvested and passed at least five consecutive days *in vitro*. All strains maintained their colony phenotype (data not shown). Together, these findings show that the recombinant *hsdS* variants were genetically “locked” in the opaque (strains A and B) or transparent (strains C, D, E and F) phase.

### The phase phenotype did not affect adherence to human epithelial cells.

In a recent study, phase-locked strains in *S. pneumoniae* ST556 (representing a serotype 19F background) were shown to differ in their levels of adherence to immortalized epithelial cells; in particular, the opaque phase had reduced adherence to human lung (A549) and nasopharyngeal (Detroit 562) epithelial cell lines (20). This is consistent with many previous studies using enriched populations (9, 14, 28–30). To determine if our phase-locked strains had similar differences in adherence, bacterial adherence studies were performed essentially as described previously (30, 31) using immortalized human lung (A549) and human bronchoepithelial (HPE14 and CFBE-4o) cell lines. Surprisingly, we did not observe significant differences in adherence (data not shown).

### Opaque-phenotype variants had diminished biofilm formation.

**FIG 4:**
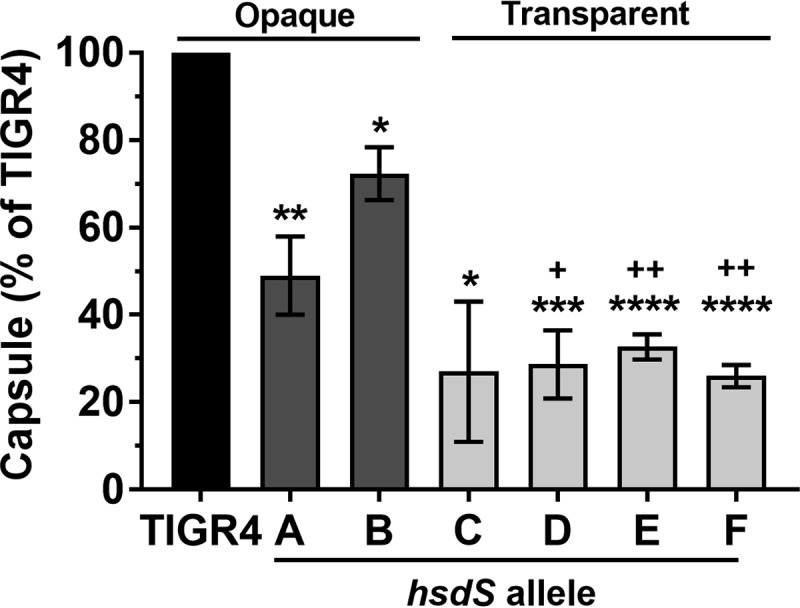
TIGR4 *hsdS* variants expressed lower levels of capsule than TIGR4. Indirect binding ELISAs were performed on heat-killed pneumococcal cultures normalized to the same density. Results were normalized to TIGR4, and data are represented as means ± SEM of results from three independent cultures of each strain. *, *P* < 0.05; **, *P* < 0.01; ***, *P* < 0.001; ****, *P* < 0.0001 (versus TIGR4). +, *P* < 0.05; ++, *P* < 0.01 (versus strain B). TIGRJS was used as a negative control. A two-tailed unpaired parametric *t* test determined statistical significance.

**FIG 5:**
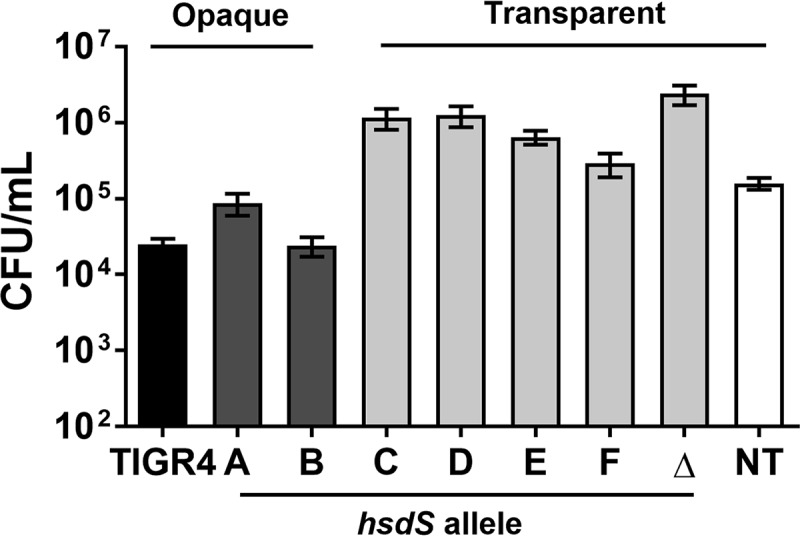
Biofilm formation was affected by phase phenotype. Pneumococci were seeded at 5 × 10^5^ CFU/ml in TSB plus catalase (2,500 U/ml) into 24-well plates, and viability was assessed at 24 h. Data are represented as means ± SEM and were pooled from results from three independent experiments, with three biological replicates per experiment. A two-tailed unpaired parametric *t* test determined statistical significance. *, *P* < 0.05; **, *P* < 0.01; ***, *P* < 0.001; ****, *P* < 0.0001 (versus TIGR4). +, *P* < 0.05; ++, *P* < 0.01 (versus strain B).

Opaque colony variants have decreased adherence to host cells, animal nasal surfaces, and plastic presumably due to overexpression of capsular polysaccharide, which can mask subcapsular adhesins (9, 30, 32). To determine whether our phase-locked mutant strains differed in capsule expression, we performed enzyme-linked immunosorbent assays. We found that the *hsdS* variants produced less capsule than *S. pneumoniae* TIGR4 and that the transparent variants produced less capsule than the opaque strains (Fig. 4). To determine whether the strains differed in their ability to form biofilms *in vitro*, we performed static biofilm assays essentially as previously described (30). Total biomass and bacterial viability within biofilms were assessed by crystal violet staining and by conventional colony counting, respectively, at the 4 h and 24 h time points. Although no difference in the levels of biomass was detected at either time point (data not shown), we observed diminished bacterial counts within biofilms for the opaque strains (*S. pneumoniae* TIGR4, A, and B) after 24 h (Fig. 5). On the basis of these results, we conclude that opaque variants have a diminished capacity to survive within mature biofilms.

### Colonization, persistence, and virulence of phase-locked mutants.

**FIG 6:**
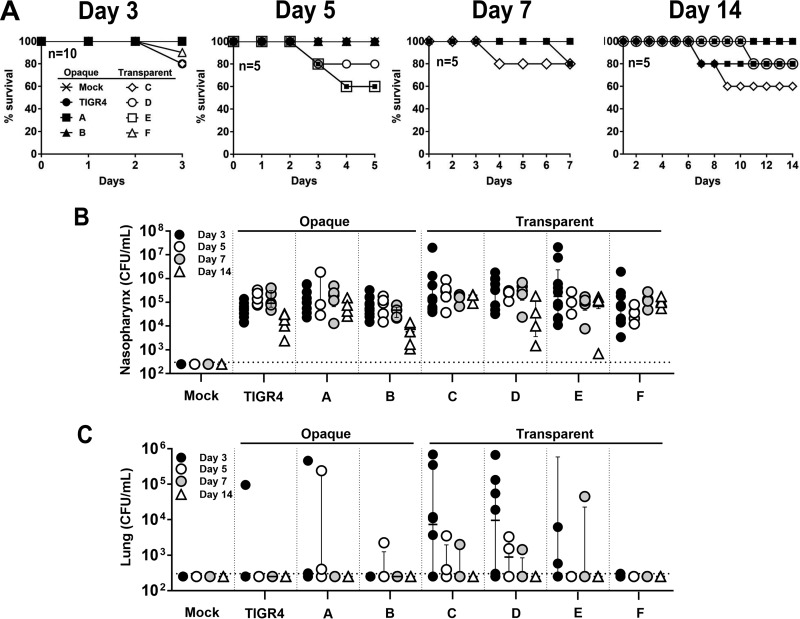
Comparison of levels of mouse virulence of single-allele *hsdS* mutants. (A) Female BALB/c mice (5/group) were infected intranasally with 10^6^ CFU. A Kaplan-Meier curve shows the percentage of survival of each group. (B and C) At 3, 5, 7, and 14 days postinfection, the nasopharynx (B) and right lung (C) were harvested and processed for bacterial counting. The dashed line represents the limit of detection. Vertical dashed lines were added to better visualize data. Data are shown with interquartile range.

Nasal colonization studies were conducted with *S. pneumoniae* TIGR4; opaque variants A and B; and transparent variants C, D, E, and F. Briefly, BALB/c mice were infected intranasally with ~10^6^ CFU, and at 3, 5, 7, and 14 days postinfection, groups of mice were euthanized and the nasopharynx and right lung lobe were excised, homogenized, and plated on blood agar containing gentamicin (4 μg/ml) for bacterial counts. All mice infected with the parental *S. pneumoniae* TIGR4 strain showed nasal carriage that did not result in bacteremia, which is consistent with our prior infection studies. Remarkably, only mice infected with transparent strains C and D appeared visibly sick and had considerable weight loss (data not shown). The mortality rates differed for each of the phase-locked strains (Fig. 6A).

All strains maintained nasal colonization for 14 days (Fig. 6B). The opaque strains had a small colonization defect on day 3 and a reduced bacterial load on day 14. Interestingly, transparent strains C and D were more virulent and caused the majority (61.5%) of the lung infections (Fig. 6C), particularly at the early time points. The levels of the lung infections decreased steadily, with no lung infections detected on day 14. Although strain F had stable colonization, infection with this strain did not progress to lung infection (Fig. 6B and C). On the basis of these results, we conclude that *S. pneumoniae* phase variation mediated by *hsdS* alleles C and D had a significant impact on pneumococcal colonization, persistence, and virulence.

### Phase-locked variants maintained phenotype for 2 weeks *in vivo*.

**FIG 7:**
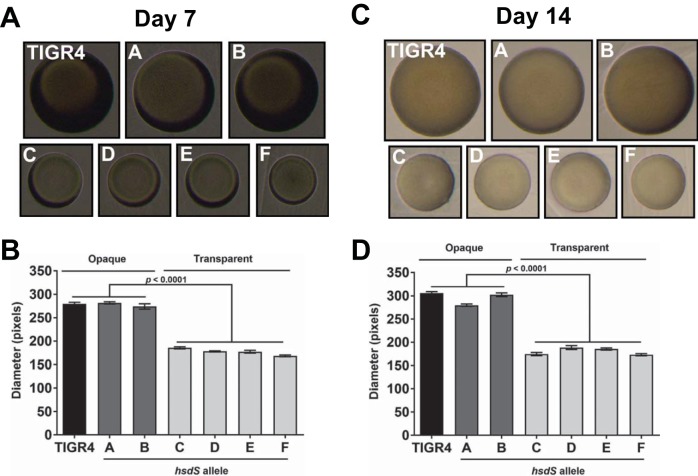
Colony phenotypes were stable on passage. Nasal tissue homogenates were plated on translucent agar and grown for 18 to 20 h. (A and C) Individual colonies were photographed, and representative images are shown. (B and D) Colony diameters were measured with ImageJ.

Previous *in vitro* studies found that serial passage of the phase-locked *hsdS* mutants *in vitro* did not significantly affect colony size or phenotype (data not shown). Therefore, on days 7 and 14, nasal and lung tissue homogenates from the animal experiments were plated on Trypticase soy agar containing gentamicin for determinations of colony phenotype. At least 50 individual colonies were imaged (Fig. 7A and C), and their diameters were measured. The average colony diameters were nearly identical for the *S. pneumoniae* TIGR4, A, and B strains and for transparent strains C, D, E, and F (Fig. 7B and D). The opaque and transparent colony sizes differed by ~39%. These findings showed that all strains tested were phase-locked and could maintain their colony phenotype after passage in an animal for 14 days.

### HsdS-dependent changes in gene expression.

Bacterial DNA methylation can result in epigenetic changes that can affect gene expression. Previous studies of *S. pneumoniae* D39 and *S. pneumoniae* ST556 and their phase-locked variants determined that each *hsdS* allele was associated with distinct DNA methylation patterns and with altered virulence gene expression (19, 20). For example, the *S. pneumoniae* D39 variant that harbored *hsdS* allele B had reduced expression of the *luxS* gene and the capsular polysaccharide synthesis operon (19). In this study, RNA sequencing was used to determine whether our panel of *hsdS* variants had unique transcriptomes. Data sets were deposited in the NCBI GEO database (accession number GSE103364).

Changes in gene expression compared to that seen with *S. pneumoniae* TIGR4 *hsdS* deletion mutant MBO15 were detected for all strains. The data were searched for a set of genes that had similar altered expression between the transparent (C, D, E, and F) and opaque (A and B) strains. Although no such genes were detected for the transparent strains, opaque strains A and B had reduced expression of 10 genes, with about half being associated with glutamine biosynthesis and ABC transporters (Table 2). Next, each strain was examined for a set of genes that had altered expression only in that strain. *S. pneumoniae* TIGR4 had 411/817, A had 15/22, B had 16/27, C had 18/30, D had 2/27, E had 1/2, and F had 0/0 genes upregulated. Notably, strain A had increased expression of a tryptophan synthesis operon (genes *SP_1811* to *SP_1817*) known to be involved in *S. pneumoniae* TIGR4 virulence (33). Strain C had increased expression of three genes (*SP_1383*, *SP_2175*, and *SP_2176*) known to be involved in d-alanylation of cell wall teichoic acids. Finally, strain D had reduced expression of six genes (*SP_0957*, *SP_2002* and *SP_2003*, and *SP_1796* to *SP_1798*) that encoded ABC transporters. Strains B and E had differential gene expression whereas strain F had no altered gene expression compared to strain MBO15. Thus, we concluded that the six *hsdS* alleles altered gene expression of potential virulence factors in the different phase-locked strains. Four genes were selected for validation with real-time PCR (Table S4). While most of the transcriptome sequencing and reverse transcription-PCR data were similar, incongruent data were observed for strains TIGR4 (genes *SP_0148* and *SP_2176*), E (*SP_0148* and *SP_2176*), and F (*SP_1797*).

**TABLE 2:** Gene expression differences between strains

HsdS allele	Gene ORF^a^	Gene	log_2_ fold change for allele^b^:							Product
			A	B	C	D	E	F	TIGR4	
B and C	SP_0176	*ribAB*	0.9	1.0	1.7	−0.4	0.6	0.3	0.4	Bifunctional 3,4-dihydroxy-2-butanone 4-phosphate synthase/GTP cyclohydrolase II
	SP_2269	*SP_2269*	0.6	1.3	1.1	0.5	0.0	−0.6	0.0	tRNA-Val
										
A and B	SP_RS02430	*SP_RS02430*	−1.5	−1.1	0.1	−0.8	0.2	−0.1	0.3	Hypothetical protein
	SP_0116	*SP_0116*	−1.1	−1.2	−0.9	−0.6	−0.1	0.1	−0.1	Hypothetical protein
	SP_0148	*SP_0148*	−2.9	−2.4	−0.4	0.1	0.4	0.2	0.0	Amino acid ABC transporter substrate-binding protein
	SP_0149	*SP_0149*	−2.0	−1.9	−0.3	−0.1	−0.1	−0.1	0.5	Lipoprotein; *O*-sialoglycoprotein endopeptidase
	SP_0150	*SP_0150*	−1.9	−1.4	−0.1	−0.2	−0.2	−0.1	0.3	Peptidase M20
	SP_0266	*glmS*	−1.3	−1.4	−0.2	−0.8	−0.6	−0.8	0.6	Glutamine–fructose-6-phosphate aminotransferase
	SP_0620	*SP_0620*	−1.9	−1.2	0.0	0.1	0.1	0.0	−0.9	Glutamine ABC transporter substrate-binding protein
	SP_1064	*SP_1064*	−1.2	−1.1	0.1	−0.7	−0.1	−0.2	−1.0	Transposase
	SP_1460	*SP_1460*	−1.3	−1.4	−0.5	0.0	0.5	0.3	−0.4	Amino acid ABC transporter ATP-binding protein
	SP_1622	*SP_1622*	−1.4	−1.2	0.1	−0.8	−0.2	−0.2	−1.0	Transposase, IS*200* family
	SP_1865	*pepA*	1.0	1.1	0.5	0.8	0.3	0.1	−0.4	Glutamyl aminopeptidase
										
A	SP_1811	*trpA*	1.2	0.8	−0.22	−0.2	−0.1	−0.2	0.0	Tryptophan synthase subunit alpha
	SP_1812	*trpB*	1.4	0.8	−0.23	−0.1	−0.2	−0.2	−0.3	Tryptophan synthase subunit beta
	SP_1813	*trpF*	1.3	0.7	−0.25	0.0	−0.3	−0.3	−0.6	N-(5′-phosphoribosyl)anthranilate isomerase
	SP_1814	*trpC*	1.5	0.7	−0.20	0.2	−0.3	−0.2	−0.6	Indole-3-glycerol-phosphate synthase
	SP_1815	*trpD*	1.5	0.6	−0.18	0.2	−0.4	−0.3	−0.7	Anthranilate phosphoribosyltransferase
	SP_1816	*trpG*	1.4	0.4	−0.25	0.1	−0.6	−0.5	−0.8	Glutamine amidotransferase
	SP_1817	*trpE*	1.8	0.4	0.26	0.5	−0.6	−0.4	−0.7	Anthranilate synthase component I
										
B	SP_2237	*comC2*	−0.2	−1.4	0.25	−0.9	−0.8	−0.5	0.3	Competence-stimulating peptide type 2
										
C	SP_1383	*alaS*	0.3	0.2	1.00	0.7	0.2	0.0	−0.1	Alanine-tRNA ligase
	SP_2175	*dltB*	0.9	0.6	1.16	0.4	0.1	−0.1	−1.0	d-Alanyl-lipoteichoic acid biosynthesis protein DltB
	SP_2176	*dltA*	0.9	1.0	1.24	0.7	0.2	−0.1	−0.1	d-Alanine–poly(phosphoribitol) ligase
										
D	SP_0957	*SP_0957*	−0.6	0.5	−0.52	−1.2	0.4	0.2	−0.1	Peptide ABC transporter ATP-binding protein
	SP_0978	*coiA*	−0.6	0.3	−0.57	−1.5	−0.2	−0.2	0.0	Competence protein CoiA
	SP_2003	*SP_2003*	0.3	0.2	−0.35	−1.1	0.2	0.0	−0.9	Multidrug ABC transporter ATP-binding protein
	SP_2002	*SP_2002*	0.0	0.3	−0.40	−1.3	0.1	−0.1	−0.9	Multidrug ABC transporter permease
	SP_1796	*SP_1796*	0.5	0.1	−0.36	−2.2	0.8	0.2	−0.7	ABC transporter substrate-binding protein
	SP_1797	*SP_1797*	0.1	−0.2	−0.49	−2.3	0.7	0.5	−0.8	Sugar ABC transporter permease
	SP_1798	*SP_1798*	0.5	−0.3	−0.22	−2.2	0.9	0.2	−0.2	ABC transporter permease

a ORF, open reading frame.

b Data are shown as log_2_ fold change versus MBO15.

## DISCUSSION

Research in the field of pneumococcal phase variation has been hampered by the lack of availability of phase-locked strains. Recent genetic findings revealed that site-specific recombination of the *hsdS* DNA methylase targeting subunit in a type I restriction-modification locus could result in six distinct phase-locked subpopulations with different colony opacity phenotypes and gene expression characteristics (19, 20). In this study, we used a genetic approach to produce six phase-locked subpopulations of *S. pneumoniae* TIGR4 and to examine their colony sizes, ability to form biofilms, attachment to host cells, and persistence in nasal colonization. Analysis of the phenotypes could provide insights into the mechanism(s) (e.g., contribution of phase-specific virulence factors) that facilitates the adaptation of pneumococci to different host environments.

This study demonstrated that recombinations of the *hsdS* gene in the *S. pneumoniae* TIGR4 type I restriction-modification system resulted in six distinct bacterial population derivatives that were 100% phase-locked in the opaque (strains A and B) or transparent (strains C, D, E, and F) phenotype. Importantly, our *hsdS* variants maintained their phenotype over multiple consecutive passages *in vitro* and for 2 weeks *in vivo*. Unlike those reported for *S. pneumoniae* D39 *hsdS* derivatives (19), our colony phenotype determination did not detect mixed bacterial populations for any *S. pneumoniae* TIGR4 *hsdS* derivative. These findings were supported by another study which produced the same six recombinant *hsdS* derivatives in several different strain backgrounds, including *S. pneumoniae* TIGR4, and determined that they were 100% phase-locked (20). One major difference between our studies was that the opaque phenotype was linked only to expression of *hsdS* allele E in reference 20 but was linked to *hsdS* alleles A and B in this study. Another major difference was that their *S. pneumoniae* TIGR4 strain and its six *hsdS* derivatives all produced 100% opaque colonies (20). Although this supported the finding that our *S. pneumoniae* TIGR4 was 100% opaque, it is unclear why our results differed in colony phenotype determinations. One explanation for these differences could simply be the genetic and phenotypic diversity of the strains (34, 35). Epigenetic changes in gene expression could alter certain surface-expressed moieties that may be missing in the genomes of different strains. Despite these differences, we can conclude that each *hsdS*-locked genotype limited intrastrain variation to a single phenotype in this study.

Transparent variants have been shown to have increased biofilm formation and adherence to epithelial cells (9, 14, 28–30, 32). While we saw no differences in adherence to immortalized epithelial cells or in the early stages of biofilm formation, the opaque phase-locked variants (A and B) showed a significant decrease in bacterial counts within biofilms at later stages. It is possible that the opaque strains had altered expression of surface factors that altered their adherence to plastic surfaces. Although the phase phenotype affected biofilm formation and viability, it played a less significant role in adherence to host cells and stable colonization.

Bacterial virulence has been linked to opaque colony phenotypes (8). On the basis of previous studies, we expected that the opaque strains would have a colonization defect but increased invasiveness. Unexpectedly, we found that transparent phase-locked strains C and D were hypervirulent and caused the majority of lung infections. This finding does not seem to fit with the results seen in the field, where transparent variants are considered generally nonvirulent. One explanation may be that it is difficult to compare a phase-locked strain population to heterogeneous populations. Another explanation may be that the phase-locked strains differed in gene expression.

It has been demonstrated that *hsdS* derivatives in *S. pneumoniae* D39 (19) and six other strains (20) had distinct genome methylation patterns with the exact same methylation sequences for each allele. For example, both studies found that *hsdS* genes encoding TRD 1.1 and TRD 2.2 methylated the same adenine in the motif 5′ CRAANNNNNNNNCTT 3′. DNA methylation is known to result in epigenetic changes that can alter gene expression. We performed RNA-seq studies to help to identify potential virulence factors that may differ in each phase. We found that all strains in our study had differential gene expression compared to *S. pneumoniae* TIGR4 *hsdS* locus deletion strain MBO15. ABC transporter gene *SP_0148* was the most highly downregulated gene in opaque strains A and B. Interestingly, deletion of *SP_0148* in *S. pneumoniae* TIGR4 was shown to attenuate virulence in pulmonary infections (36). This may help explain why opaque variants A and B were less virulent than the transparent strains. Of the 18 genes upregulated only in variant C, 3 (*SP_1383*, *SP_2175*, and *SP_2176*) were involved in d-alanylation of cell wall teichoic acids. This type of cell wall modification has been shown to increase Gram-positive bacterial resistance to antimicrobial peptides (via an increase in the net surface positive charge) (37). This finding may help to partially explain why variant C was so virulent *in vivo*. Notably, variant D showed reduced expression of six ABC transporter genes (*SP_0957*, *SP_2002* and *2003*, and *SP_1796* to *SP_1798*). The *S. pneumoniae* TIGR4 genome encodes 73 ABC transporters (38), and mutation of genes *SP_1796* to *SP_1798* in TIGR4 was shown to not affect virulence *in vivo* (36). Therefore, reduced expression of that ABC transporter in this study may have had only a minimal effect during the course of the infection.

One potential limitation in this study was that we created single-allele *hsdS* derivatives of only one strain, *S. pneumoniae* TIGR4. However, with our recombinant *hsdS* constructs in hand, it will be straightforward to produce variants in other pneumococcal backgrounds. Another limitation is that we did not assess DNA methylation patterns for each variant. On the basis of our findings, we hypothesize that, as has been shown in previous studies (19, 20), the DNA methylation patterns likely differ in the recombinant *hsdS* variants.

Since we have phase-locked strains that differ in colony size, biofilm formation, and persistence *in vivo*, it would be interesting to investigate the contribution of specific virulence factors to each phenotype. By deleting and complementing various factors within the phase(s) in which they are predominantly expressed, we have the potential to more directly and specifically address contributions to virulence or virulence-related phenotypes. Further, since genes *glnA* and *hsdM* are conserved among different pneumococcal strains, the same genetic constructs used in this study can be transformed into multiple genetic backgrounds. This would be useful to study the contribution of certain virulence factors in a serotype-specific manner.

Epigenetic phase variation associated with variable expression or the specificity of DNA methylase is emerging as a common theme for generation of phenotypic diversity in bacterial populations. Our construction of single-allele *hsdS* mutant substrains offers the possibility of refining molecular pathogenesis work by specifically studying virulence factors within one, or a few, phase types. This study demonstrated that the phase-locked *S. pneumoniae* TIGR4 *hsdS* strains offer a useful model for the study of the contribution of colony phenotype to multiple aspects of host-pathogen interactions.

## MATERIALS AND METHODS

### Pneumococcal culture conditions.

A list of all pneumococcal strains used in this study is provided in Table 1 in the supplemental material. Bacteria were cultured in a humid atmosphere at 37°C in 5% CO_2_. Pneumococci were grown on tryptic soy agar (Difco, BD Diagnostics) supplemented with 5% sheep’s blood (Hemostat) and 4 µg/ml gentamycin (Sigma) or in Todd-Hewitt broth (Difco, BD Diagnostics) supplemented with 0.5% yeast extract (THY medium; Difco, BD Diagnostics) at 37°C. Freezer stocks were made in 18 to 20% glycerol.

### Construction of six recombinant *hsdS* alleles.

The recombinant *hsdS* alleles were produced via overlap extension PCR using *S. pneumoniae* TIGR4 genomic DNA, primers F1-F10 and R1-R8 (see Table S2 and Fig. S1 in the supplemental material), and TaKaRa *Ex Taq* polymerase (Clontech). The primer pairs, DNA targets, and expected PCR amplicon sizes are shown in Table S3. For example, to create *hsdS* allele A (TRD 1.1 and 2.1), PCR amplicon “A1” (gene target TRD 2.1) was produced with primer pair F4/R3 (~0.5 kb), and “A2” (IR2R through *hsdM*) was produced with primer pair F8/R8 (~2.3 kb). The “A1” and “A2” PCR amplicons were mixed together (50 to 100 ng each) and amplified with primer pair F4/R7 (~2.6 kb) to produce the final *hsdS* allele, allele A. Recombinant *hsdS* alleles B through F were similarly produced with their specific primer pairs. All PCR amplicons and final constructs were gel purified to reduce the possibility of parental chromosomal contamination during the overlap extension PCR procedure.

10.1128/mSphere.00386-17.1 FIG S1  Schematic diagram showing primer binding locations and gene annotation for each TRD. The TIGR4 *hsdS* locus (GenBank accession number AE005672.3; DNA positions 483927 to 494129) and the TIGRJS Janus cassette locus are shown. For clarity, individual genes and the DNA target recognition domains were annotated. Primer names (F1-F11 and R1-R9), their binding locations (circle symbols), and target *hsdS* alleles (in parentheses) are shown. Δ, *hsdS* deletion strain MBO15. Download FIG S1, EPS file, 2.2 MB. Copyright © 2017 Oliver et al. 2017 Oliver et al. This content is distributed under the terms of the Creative Commons Attribution 4.0 International license.

### Construction of recombinant *hsdS* genetic constructs.

The recombinant *hsdS* allele PCR amplicons (A through F) were cloned into plasmid pCR2.1 (Invitrogen) and then transformed into *E. coli* strain DH5α. *E. coli* bacteria were cultured in Luria-Bertani (LB) agar or broth (Difco, BD Diagnostics) supplemented with the appropriate antibiotic (kanamycin [30 µg/ml] or spectinomycin [100 µg/ml]). Purified plasmids (Qiagen) were sequenced (Eton Biosciences, Durham, NC), linearized, and ligated to the *aad9* spectinomycin resistance gene, previously digested and gel purified from plasmid pSpecR (pCR2.1 containing *aad9*) (39). The *aad9*-*hsdS* constructs were transformed into *E. coli* DH5α. Purified plasmids were linearized, ligated to the upstream flanking sequence (*S. pneumoniae* TIGR4 *glnA* through *SP_0504*), and transformed into *E. coli* DH5α. Plasmids containing the final constructs were sequenced and stored at −20°C until used.

### Pneumococcal transformations.

All transformations were performed with 100 ng of donor DNA as previously described (27, 40) and were cultured on Trypticase soy agar or in THY broth containing the appropriate antibiotic (kanamycin [100 µg/ml] or spectinomycin [100 µg/ml]). A *S. pneumoniae* TIGR4 *hsdS* deletion mutant (named MBO15) was produced by replacing all the genes located between *SP_0504* and *hsdM* (DNA positions 485939 through 490690) with a Janus cassette as described previously (26, 27). The primers used to create this *hsdS* knockout construct are listed in Tables S2 and S3 (see also Fig. S1). Confirmation by DNA sequence analysis of the relevant region of genomic DNA revealed no unexpected mutations. Plasmids containing each recombinant *hsdS* allele were linearized with XhoI (New England Biolabs) and transformed into MBO15 to create strains MBO20 to MBO25. Spectinomycin-resistant clones were sequenced from *glnA* to *hsdM*, and their sequences were submitted to GenBank (accession numbers MF927926 through MF927932).

10.1128/mSphere.00386-17.4 TABLE S3  Primer pairs used to create recombinant *hsdS* genes. The six predicted *hsdS* genes (first column) were produced via overlap extension PCR. Primer pairs (third column) targeted specific gene locations in TIGR4 (fourth and fifth columns) to create PCR amplicons (second column) of known sizes (sixth column). The individual PCR amplicons were “stitched” together to create recombinant *hsdS* genes. Download TABLE S3, DOCX file, 0.02 MB. Copyright © 2017 Oliver et al. 2017 Oliver et al. This content is distributed under the terms of the Creative Commons Attribution 4.0 International license.

10.1128/mSphere.00386-17.5 TABLE S4  RNA-seq data were validated with RT-PCR. The expression levels of genes *SP_0148*, *SP_1797*, and *SP_2176* were validated by RT-PCR. All data shown in the table represent the total fold change seen with each gene relative to MBO15. Positive values indicate increased expression, while negative values indicate reduced expression. RT-PCR data were normalized to endogenous control gene *gryB*. Download TABLE S4, DOCX file, 0.02 MB. Copyright © 2017 Oliver et al. 2017 Oliver et al. This content is distributed under the terms of the Creative Commons Attribution 4.0 International license.

### Colony phase determination.

Thawed freezer vials were diluted in phosphate-buffered saline (PBS) and grown on Trypticase soy agar supplemented with catalase (2,500 U/ml) for 18 to 20 h. Bacterial colony phenotypes were assessed by phase-contrast microscopy under oblique light. Colonies were visualized with a Fisher Micromaster microscope (magnification, ×40), and their phenotypes were photographed with a digital camera (Basler) and its associated imaging program (Basler Pylon V4.0.1.3425). ImageJ V1.49 was used to measure the diameter of individual colonies. Strains were subjected to *in vitro* passage for at least five consecutive days by harvesting all colonies from a culture plate, washing in PBS, serially diluting, and plating onto fresh agar. The viability and colony size of the *hsdS* variants were not affected by exposure to either of the antibiotics spectinomycin (100 µg/ml) and gentamicin (4 µg/ml). Each of these experiments was repeated at least five times.

### Biofilm formation.

Thawed freezer vials were diluted to 5 × 10^5^ CFU/ml in tryptic soy broth (Difco, BD Diagnostics) supplemented with 2,500 U/ml catalase (Worthington Biochemical Corporation, Lakewood, NJ) and seeded (1 ml/well) into 24-well polystyrene plates (Corning Costar, Cambridge, MA). Plates were incubated at 37°C in 5% CO_2_ for 24 h. Supernatants were carefully removed and replaced with 1 ml PBS. Biofilms were scraped into solution, serially diluted in PBS, and plated on blood agar for counting viable bacteria. The experiment was repeated three times.

### ELISA.

Relative levels of capsule expression were quantified using direct binding enzyme-linked immunosorbent assays (ELISAs). Three independent cultures of each strain were grown in THY medium and harvested at an optical density at 600 nm (OD_600_) of 0.400 ± 0.05. Cultures were heat killed (56°C for 20 min) and centrifuged (12,000 rpm for 5 min), and the pellets were suspended in PBS to the original culture volume. Each sample was normalized to an OD_600_ of 0.03 ± 0.05 in PBS and stored at 4°C until used. The normalized samples were serially diluted 3-fold in PBS (pH 7.22) and coated on microtiter plates (Corning CoStar catalog no. 9017; 100 µl/well) overnight at 4°C in a humidified container. Coated plates were washed three times (wash buffer: 1.26 mM NaCl, 200 µM Trizma base, and 30 µM Trizma HCl at pH 7.22 ± 0.02) and blocked with 1% bovine serum albumin–PBS for 1 h at 37°C. Following another three washes, bound bacteria were detected with polyclonal type 4 antiserum [Statens Serum Institut catalog no. 16747(SS)] previously adsorbed against the nonencapsulated *S. pneumoniae* TIGRJS strain at a 1:250 dilution in antibody buffer (PBS [pH 7.22 ± 0.02], 0.05% Tween 20) for 1 h at room temperature. Following another three washes, bound antibodies were detected with goat anti-rabbit immunoglobulin conjugated to alkaline phosphatase (SouthernBiotech catalog no. 4010-04) used at a 1:3,000 dilution in antibody buffer for 1 h at room temperature. Following a final three washes, p-nitrophenyl phosphate substrate (Thermo Scientific catalog no. 34045 and 34064) cleavage was detected at OD_450_ using a BioRad Benchmark Plus spectrometer and data were collected using microplate Manager V5.1 software. Data were analyzed using GraphPad Prism V7.02.

### Antiserum adsorption.

A 0.5-liter THY culture of the capsule-negative *S. pneumoniae* TIGRJS strain was harvested at an OD_600_ of 1.0, heat killed (56°C for 20 min), and centrifuged (12,000 rpm for 10 min), and the pellet was suspended in 10 ml PBS. Non-capsule antibody adsorption was achieved by diluting the polyclonal antiserum 1:10 in PBS and then adding an equal volume of *S. pneumoniae* TIGRJS pellet suspension. The mixture was incubated overnight at 4°C under shaking conditions. The bacterium-serum mixture was centrifuged (12,000 rpm for 5 min), and the supernatant was filtered and stored at 4°C until use.

### Mouse infection studies.

Female BALB/c mice (Jackson Laboratories; 6 to 8 weeks old; 5 per group) were anesthetized with isoflurane and infected intranasally with ~10^6^ CFU of pneumococci in 20 µl of THY or with an equal volume of vehicle control. Bacterial counts were confirmed by plate counting. At 3 days, 5 days, 7 days, and 14 days postinfection, mice were euthanized and the nasopharynx and right lung were excised and homogenized in 3 ml and 1.5 ml PBS, respectively. The left lung was saved for histopathology. Tissue homogenates were serially diluted in PBS and plated on blood agar containing gentamicin (4 μg/ml) for bacterial counting and on Trypticase soy agar containing gentamicin (4 μg/ml) for colony imaging. Plates were incubated for 18 to 20 h at 37°C with 5% CO_2_. All animal experiments were approved by the University of Alabama at Birmingham Institutional Animal Care and Use Committee (Animal Project number IACUC-20589).

### Gene expression analyses.

For each pneumococcal strain, three independent cultures were grown in THY medium to mid-log phase (OD_600_ of 0.8), centrifuged (12,000 rpm for 5 min), and resuspended in 1 ml PBS containing lysozyme (2 mg/ml). Total RNA was extracted using a NucleoSpin RNA II kit (Macherey-Nagel, Germany) per the manufacturer’s recommendations. Frozen (−80°C) RNA samples were sent to the UAB Heflin Center for Genomic Sciences for RNA-seq analyses using an Illumina HiSeq 2500 platform (Illumina). The rRNA was removed from the total bacterial RNA using ribosome reduction for Gram-positive bacteria (ArrayStar, Rockville, MD). The purified RNA was then processed using a SureSelect strand-specific RNA-Seq library prep kit from Agilent Technologies (San Diego, CA) following the manufacturer’s protocol. The resulting libraries were quantitated with quantitative PCR (Kapa Biosystems, Woburn, MA), and the size distributions of the insertions were checked using an Agilent 2100 BioAnalyzer and a high-sensitivity DNA chip. The library concentration was normalized to 2 nM, and sequencing on a HiSeq 2500 platform with paired-end 50-bp sequences was performed under standard conditions. Sequencing generated 61-bp single-end RNA-Seq reads with an average depth of 16.8 + 2 M reads. The sequencing depth translated to ~475× coverage (based on the length of the *S. pneumoniae* TIGR4 genome [2.1 MB]). The quality of FASTQ files were checked using tool FASTQC. Reads were mapped to the reference *S. pneumoniae* TIGR4 genome (accession number NC_003028.3) using Bowtie2 (96% reads mapped), and transcript abundance was calculated using the summarizeOverlaps function in R package GenomicAlignments. Finally, differential expression analyses were carried out using R package DeSeq2. All data were compared to data from strain MBO15 (an *S. pneumoniae* TIGR4 *hsdS* deletion variant). The RNA-seq data were deposited in the NCBI GEO database (accession number GSE103364).

### Real-time PCR.

Target genes *SP_0806*, *SP0148*, *SP_1797*, and *SP2176* were selected for validation. Custom oligonucleotides and probes conjugated to 6-carboxyfluorescein were designed using the IDT PrimerQuest Tool (Table S3). Real-time PCR was performed using a TaqMan RNA-to-Ct 1-Step kit (Applied Biosystems catalog no. 4392938) in triplicate 20 μl reaction mixtures containing 2× master mix, a 900 nM concentration of each primer, 100 nM fluorescently labeled probe, and RNA at a final concentration of 10 ng/μl. Amplification and detection were performed using a QuantStudio 3 real-time PCR system (Thermo Scientific). PCR conditions were 48°C for 2 min and 95°C for 10 min, followed by 40 cycles of 95°C for 15 s and 60°C for 1 min. ROX1 was used as the passive reference dye. The transcription level of each gene was normalized to the *gyrB* reference gene (41), and the results were analyzed using the comparative threshold cycle (*C*_*T*_) method. The experiments were repeated twice.

### Accession number(s).

The sequences for strains MBO15 (Δ*hsdS*), MBO20 (*hsdS* allele A), MBO21 (*hsdS* allele B), MBO22 (*hsdS* allele C), MBO23 (*hsdS* allele D), MBO24 (*hsdS* allele E), and MBO25 (*hsdS* allele F) were submitted to the GenBank database under accession numbers MF927926 through MF927932, respectively. The RNA-seq data for this study are available in the Gene Expression Omnibus database (http://www.ncbi.nlm.nih.gov/geo/) under accession number GSE103364.
