# Supplementary material for: Streptococcus pneumoniae TIGR4 Phase-Locked Opacity Variants Differ in Virulence Phenotypes
Source: mSphere. 2017 Nov 15;2(6):e00386-17. doi: 10.1128/mSphere.00386-17 (PMC5687919; doi:10.1128/mSphere.00386-17)
Supplement: TABLE S1 [file sph006172399st2.docx]

| **Gene** | **Protein** | **DNA position** |
| --- | --- | --- |
| *SP_0502* | *gln;* type I glutamine synthetase | 483,927 - 485,274 |
| *SP_0503* | hypothetical protein | 485,370 - 485,489 |
| *SP_0504* | hypothetical protein | 485,747 - 485,884 |
| *SP_0505* | *hsdS'';* S'' subunit | 485,976 - 487,335 |
|  | IR1R (partial) | 485,976 - 486,060 |
|  | TRD 1.2 | 486,061 - 486,465 |
|  | IR2R | 486,463 - 486,775 |
|  | TRD 2.1 | 486,775 - 487,335 |
| *SP_0506* | *creX;* integrase/recombinase | 487,392 - 488,189 |
| *SP_0507* | *hsdS';* S' subunit | 488,248 - 488,799 |
|  | TRD 2.3 |  |
| *SP_0508* | *hsdS;* S subunit | 488,753 - 490,320 |
|  | TRD 2.2 | 488,753 - 489,313 |
|  | IR2R | 489,314 - 489,628 |
|  | TRD 1.1 | 489,629 - 490,045 |
|  | IR1R | 490,046 - 490,320 |
| *SP_0509* | *hsdM;* M subunit | 490,321 - 491,784 |
| *SP_0510* | *hsdR;* R subunit | 491,797 - 494,129 |
