# Supplementary material for: Streptococcus pneumoniae TIGR4 Phase-Locked Opacity Variants Differ in Virulence Phenotypes
Source: mSphere. 2017 Nov 15;2(6):e00386-17. doi: 10.1128/mSphere.00386-17 (PMC5687919; doi:10.1128/mSphere.00386-17)
Supplement: TABLE S3 [file sph006172399st4.docx]

| ***hsdS* allele** | **PCR amplicon** | **primer pair** | **DNA target(s)** | **DNA binding position** | **size (bp)** |
| --- | --- | --- | --- | --- | --- |
| **A** | A1 | F4 + R3 | TRD 2.1 | 486,775 - 487,335 | 560 |
|  | A2 | F8 + R8 | IR2R - TRD 1.1 - IR1R - *hsdM* | 489,314 - 491,629 | 2,316 |
|  | A1 + A2 | F4 + R7 |  |  | 2,663 |
| **B** | B | F6 + R8 | *hsdS* - *hsdM* | 488,753 - 491,629 | 2,877 |
| **C** | C1 | F6 + R6 | TRD 2.2 - IR2R | 488,753 - 486,481 | 873 |
|  | C2 | F3 + R2 | TRD 1.2 | 486,061 - 486,465 | 401 |
|  | C3 | F9 + R8 | IR1R - *hsdM* | 490,046 - 491,629 | 1,587 |
|  | C2 + C3 | F3 + R7 |  |  | 1,784 |
|  | C1 + C2C3 | F6 + R7 |  |  | 2,648 |
| **D** | D1 | F3 + R3 | TRD 1.2 - IR1R – TRD 2.1 | 486,061 - 487,335 | 1,275 |
|  | C3 | F9 + R8 | IR1R - *hsdM* | 490,046 - 491,629 | 1,587 |
|  | D1 + C3 | F3 + R7 |  | 486,061 - 491,416 | 2,649 |
| **E** | E1 | F5 + R4 | *hsdS"* (TRD 2.3) | 488,248 - 488,799 | 546 |
|  | E2 | F7 + R8 | IR2R - TRD 1.1 - IR1R - *hsdM* | 489,314 - 491,629 | 2,316 |
|  | E1 + E2 | R5 + R7 |  |  | 2,649 |
| **F** | E1 | F5 + R5 | *hsdS"* (TRD 2.3) | 488,248 - 488,799 | 551 |
|  | F1 | F7 + R6 | IR2R | 489,314 - 489,628 | 315 |
|  | C2C3 | F3 + R7 |  |  | 1,784 |
|  | E1 + F1 | R5 + R7 |  |  | 866 |
|  | E1F1 + C2C3 | F5 + R7 |  |  | 2,650 |
| **Δ** | Upstream flank | F2 + R1 | *glnA*-IGR | 484,980 – 485,939 | 959 |
|  | Janus cassette | F11 + R9 | antibiotic marker | N/A | ~1,300 |
|  | Downstream flank | F10 + R8 | *hsdM* | 490,690 - 491,629 | 939 |
