# Supplementary material for: Streptococcus pneumoniae TIGR4 Phase-Locked Opacity Variants Differ in Virulence Phenotypes
Source: mSphere. 2017 Nov 15;2(6):e00386-17. doi: 10.1128/mSphere.00386-17 (PMC5687919; doi:10.1128/mSphere.00386-17)
Supplement: TABLE S4 [file sph006172399st5.docx]

|  | ***SP_0148*** | | ***SP_1797*** | | ***SP_2176*** | | |  |
| --- | --- | --- | --- | --- | --- | --- | --- | --- |
| **Strain** | **RNA-seq*** | **qPCR**** | **RNA-seq** | **qPCR** | | **RNA-seq** | **qPCR** | |
| **TIGR4** | -0.040 | 1.134 | -0.768 | -1.361 | | -0.127 | 0.307 | |
| **A** | -2.895 | -2.316 | 0.106 | 0.764 | | 0.862 | 1.176 | |
| **B** | -2.373 | -2.441 | -0.199 | -0.719 | | 0.956 | 0.456 | |
| **C** | -0.375 | -0.395 | -0.487 | -0.456 | | 1.240 | 0.965 | |
| **D** | 0.114 | 0.130 | -2.316 | -1.746 | | 0.650 | 0.090 | |
| **E** | 0.361 | -0.132 | 0.744 | 0.340 | | 0.206 | -0.100 | |
| **F** | 0.206 | 0.102 | 0.500 | -0.345 | | -0.066 | -0.451 | |
